# Implementation of EACS vaccination recommendations among people living with HIV

**DOI:** 10.1007/s15010-022-01827-6

**Published:** 2022-05-06

**Authors:** Sven Breitschwerdt, Carolynne Schwarze-Zander, Ahmad Al Tayy, Julia Mutevelli, Jan-Christian Wasmuth, Jürgen K. Rockstroh, Christoph Boesecke

**Affiliations:** 1 Department of Internal Medicine I, Bonn University Hospital, Venusberg-Campus 1, 53127 Bonn, Germany; 2 German Centre for Infection Research (DZIF), Partner-site, Cologne-Bonn, Germany

**Keywords:** HIV, AIDS, Vaccination, Prevention

## Abstract

**Objectives:**

With modern combination antiretroviral Treatment (cART) a normal life expectancy among people living with HIV (PLWH) has become reality if started early enough prior to the onset of more pronounced immunodeficiency. Therefore, prevention measures against other infectious diseases among this vulnerable group have gained increased attention. Indeed, the EACS guidelines recommend vaccinations against HAV, HBV, HPV, Influenza, *Neisseria meningitidis*, *Streptococcus pneumoniae* and VZV in HIV-infected adults.

**Methods:**

All PLWH under cART attending our ID outpatient clinic between April to June 2018, were assessed during consultation for vaccination status regarding pneumococcus, Hepatitis A and B, influenza, varicella, meningococcus and HPV using a pre-defined questionnaire, vaccination certificates and medical records. In addition, the cohort database was screened for Hepatitis A and B serology and HIV surrogate markers.

**Results:**

A total of 305 PLWH (82.3% male, 17.7% female) was included, median age was 48 years (IQR 47–51). Median CD4 + T cell count was 543 (IQR 304–770), and for 297 (97.4%) PLWH CD4 + T cell count was ≥ 200/ul. The viral load was undetectable (< 40 copies/ml) in 289 (94.8%) cases. Highest vaccination rates were observed for HAV (87.4%), *Streptococcus pneumoniae* (77.4%) and Influenza (76.5%). 64.3% PLWH got vaccinated against HBV, whereas VZV vaccination only played a minor role, in the context of the high rate of cleared infections (99.0%). Lowest vaccination rates were detected for HPV (0%) and *Neisseria meningitidis* (3.0%).

**Conclusions:**

Our data suggest that vaccination rates among PLWH are higher compared to the general German population. Implementation of EACS guidelines into daily routine though is not fully executed and the need for improving vaccination rates has to be emphasized. Centrally organized vaccination registers as well as electronic medical records could be helpful tools to detect a lack of vaccination coverage and send digital vaccination reminders particularly among risk groups.

## Introduction

Since its first beginnings in the late eighteenth century vaccination has become a highly effective tool to prevent contagious diseases and their complications [1–4]. Indeed, thanks to vaccination, smallpox was completely eradicated in 1979. In times of the COVID-19 pandemic the importance and benefit of vaccines as useful and cost-effective tools to prevent morbidity and mortality from various infections has become even more obvious. Nowadays, numerous vaccines are routinely available and immunization is strongly recommended by international and national guidelines, especially for individuals with comorbidities. In Germany, the STIKO (*Ständige Impfkommission*), which is associated with the “Robert-Koch-Institut”, issues general vaccination recommendations which also apply to patients with immunodeficiencies. Despite an ongoing discussion about compulsory vaccination, no central recording of vaccination coverages has been set up in Germany to date. Moreover, in countries with mandatory vaccination such as Austria and France data about the coverage are also incomplete. In the case of Germany, most data are currently collected via local institutions (*Kassenärztliche Vereinigung*) or by surveys, especially for certain sub-populations (*Onlinebefragung von Krankenhaus-Personal zur Influenza-Impfung OKaPII* [5]). To get better insights into the vaccination coverage of the German population as well as health issues in general a population-based survey called DEGS1 (*Studie zur Gesundheit Erwachsener in Deutschland *[6]) was realized between 2008 and 2011.

According to the *“Robert-Koch-Institut”* there were 90.700 people living with HIV (PLWH) in Germany in 2019 [7], making this an important group eligible for vaccination. The underlying immunodeficiency that is caused the presence of HIV may lead to a worsened course of several vaccine-preventable diseases [8]. For PLWH the European AIDS Clinical Society (EACS) guidelines advise vaccination against Hepatitis A and B (HAV, HBV), human papilloma viruses (HPV), seasonal *Influenza*, *Neisseria meningitidis*, *Streptococcus pneumoniae and Varizella zoster virus* (VZV). Although surveillance of vaccination rates is recommended in PLWH, data on implementation of EACS recommendations into routine clinical care are sparse. Therefore, we analysed vaccination rates in an HIV outpatient clinic population at the University Hospital Bonn (tertiary care centre).

## Methods

### Study population

During the screening period between April and June 2018 every HIV-positive patient who presented to the HIV clinic for routine control, was assessed for his or her vaccination status. The evaluation included status for immunization against HAV, HBV and VZV, as well as for HPV, *Neisseria meningitidis*, *Streptococcus pneumoniae*, *Varizella zoster virus* and seasonal *Influenza*. Patients who presented to the HIV clinic for the first time or who deceased during the screening period were excluded. All patients had already been under cART at the time of inclusion. Vaccination rates were assessed using a pre-defined questionnaire during consultation as well as vaccination certificates (if available) and electronic medical records. In addition, age, gender, civil status, country of origin and route of HIV transmission as well as clinical parameters (type of HIV, HIV–RNA, and CDC classification) were documented. All participants provided written informed consent.

### Laboratory analyses

The laboratory values were determined during the screening period (April 2018–June 2018) as routine diagnostic measurements and included leukocytes, platelet count, relative and absolute CD4 and CD8 cells and the CD4/CD8 ratio. In addition to that, the CD4 nadir was collected from the medical records.

The laboratory analyses took place at the Institute of Clinical Chemistry and the Institute of Pharmacology at the University Hospital in Bonn. For the determination of the lymphocytes, flow cytometry of the Sysmex XN-Series and in combination with Fluorocell WDF agent was used.

HIV–RNA quantification was carried out at the Institute of Virology using the Abbott m2000 Real Time system for PCR, which has a lower detection level of 40 copies/ml of viral RNA.

## Results

Overall, 305 PLWH were included, 251 male (82.3%) and 54 female (17.7%). The median age was 48 years (IQR 47–51). As all participants are linked to our tertiary care centre, CD4 + T cell counts were checked on a regular basis (every 3–4 months), and current data was available. The median CD4 + T cell count at inclusion into the study was 543/ul (IQR 304–770), CD4 + T cell count was ≥ 200/ul in 297 of the patients (97.4%) and the median CD4-nadir was 260/µl. Of note, 21.3% (65/305) of the participants had initially presented with a CDC classification status C3. The rate of patients holding an immunization card at assessment was 88.5% (270/305). There was no relevant sex-difference, 226 (90.0%) of male PLWH were able to present an immunization card, compared to 44 (81.5%) of female participants (Table 1).

**Table 1:** 305 patients participated in the survey, with the majority being male (251/82.3%)

Characteristics of the participants at baseline	
Total number of Patients	305
Sex—no. (%)	
Female	54 (17.7)
Male	251 (82.3)
Age—yr	
Mean	48
Median (interquartile range)	49 (47–51)
CD4 + T cell count (/μl)	
Median (interquartile range)	543 (304–770)
CD4 + T cell count ≥ 200/μl—no (%)	297 (97.4)
CD4 + Nadir	260.1
HIV RNA copies/no. (%)	
< 40 copies/ml	289 (94.8)
> 40copies/ml	16 (5.2)
Mean copies/ml	1208.2
Immunization card upon presentation—no. (%)	270 (88.5)
Male	226 (90.0)
Female	44 (81.5%)

The mean age upon presentation was 48 years. In the case of 289 (94.8% PLWH), the viral load was not detectable, while the mean number of copies for the remaining 16 PLWH was 1208.2/ml. A CD4 + cell count of at least 200/ μl, making patients eligible for vaccination was observed in 297 cases (97.4%)

The rates for the seven different vaccinations being recommended by the EACS for PLWH showed a wide range in this survey. For 4 of them, including seasonal Influenza, Hepatitis A (HAV), Hepatitis B (HBV) and *Streptococcus pneumoniae,* a high vaccination rate was observed. On the other hand, rates *for Neisseria meningitidis*, *Varizella zoster* and Human papillomavirus were at the bottom end of the scale. Indeed, none of the participants was vaccinated against HPV and *Varizella zoster*, though for the latter one, a high percentage of cleared infections has to be mentioned (Table 2).

**Table 2:** Overview of the vaccination rates for all 7 vaccinations being recommended by EACS for HIV + people

Vaccination rates—overview	
Influenza^a^	231/302 (76.5%)
Hepatitis A^b^	
Cleared infection	28/222 (12.6%)
Vaccinated	194/222 (87.4%)
Hepatitis B^c^	
Cleared infection	70/298 (23.5%)
Chronic infection	21/298 (7.0%)
Vaccinated	133/207 (64.3%)
Neisseria meningitidis	9/305 (3.0%)
Varizella zoster	
Cleared infection	302/305 (99.0%)
Vaccinated	3/3 (100%)
Streptococcus pneumoniae	
Overall vaccination	236/305 (77.4%)
13-Valent vaccine plus polysaccharide vaccine	138/236 (58.5%)
Polysaccharide vaccine mono	33/236 (14.0%)
Human papillomavirus (HPV)	
Vaccinated	0/305 (0%)

Highest rates were noticed for Influenza, HAV, HBV and Streptococcus pneumoniae

^a^Three participants were excluded from the analysis due to an unclear status

^b^Participants with cleared infection (28) and unclear status (6) were excluded

^c^Enrolled into the analysis were 298 patients, with all participants with a cleared (70) or a chronic (21) infection on day of inquiry were considered eligible for a vaccination

**Fig. 1:**
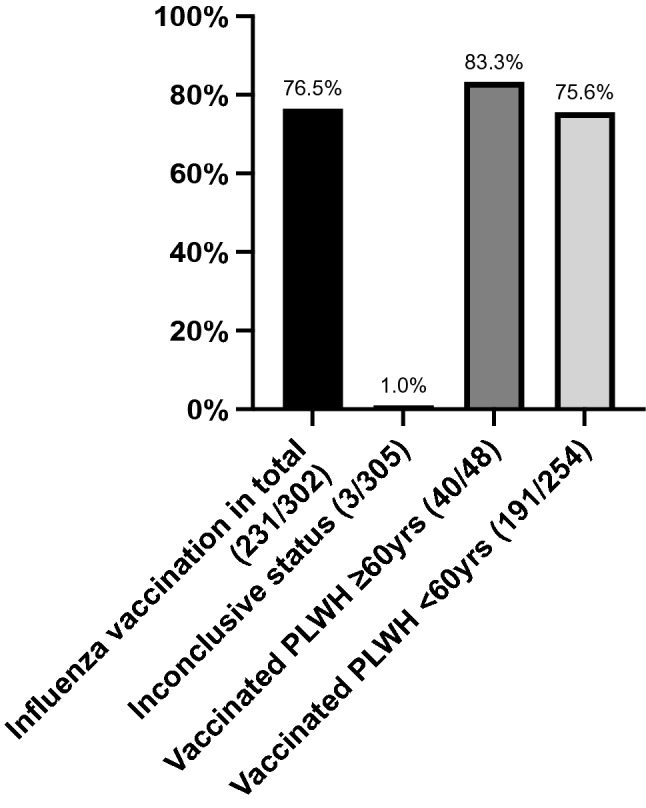
Vaccination against seasonal Influenza was high, with 76.5% of all participants having a received a current flu shot. In the subgroup of PLWH 60 years and older, vaccination coverage in our analysis was even higher (83.3%). A difference between the two genders in this subgroup was not detected

The overall vaccination rate for seasonal influenza was 76.5% (231/302). 3 participants had to be excluded from the analysis because of an inconclusive vaccination status. As the study period was April to June 2018 we assessed for vaccination during the Influenza season 2017/2018. PLWH older than 60 years showed a higher vaccination rate (83.3%) compared to participants younger than 60 years of age (75.6%) (Fig. 1). A difference between genders could not be observed.

**Fig. 2:**
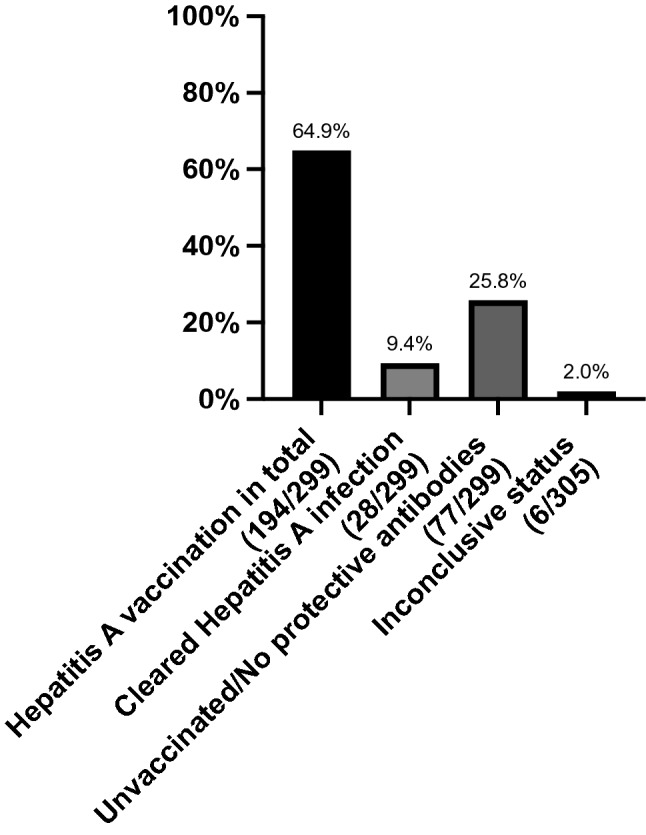
With 64.9% (194) of the participating patients, almost two thirds were vaccinated against Hepatitis A. For another 9.4% (28) antibodies could be detected after undergoing an infection. In 25.8% (77/299) of the PLWH of this study no protective antibodies, correlating with an unvaccinated status, had to be observed

The screening for HAV among our patients detected HAV antibodies in 74.2% (222/299) of cases. Only 9.4% (28/299) of the patients gained immunity after undergoing an HAV-infection, while 64.9% (194/299) of the remaining PLWH developed HAV-antibodies following HAV vaccination (Fig. 2). For 6 participants of the study data were lacking or inconclusive and neither the vaccination status nor an immune status could be obtained.

**Fig. 3:**
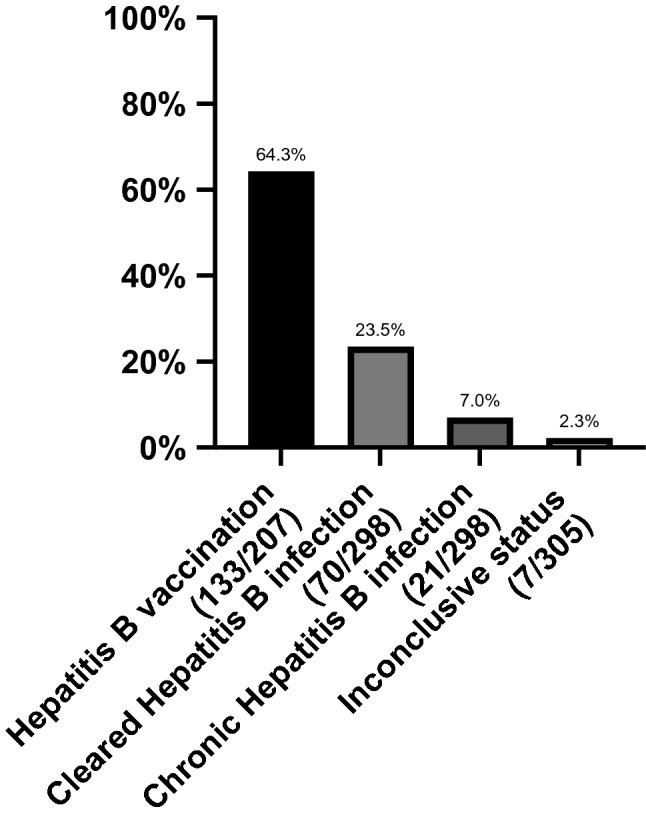
7.0% (21) of the PLWH participating in this study had a chronic HBV infection, while 23.5% (70) were able to clear an HBV infection at some earlier point. From the remaining 207 patients being eligible for vaccination, 64.3 (133) were vaccinated

Hepatitis B, known to be a common co-infection among PLWH, is also included in the EACS vaccination recommendations. In 7 patients data were lacking, so 298 could be included into analysis. 23.5% (70/298) of patients showed markers of past HBV-infection. For most of them, Anti-Hbc- and Anti-Hbs-antibodies could be detected (65/70). 1 participant had cleared an HBV-infection, but lost Anti-Hbc-Ab status, in 3 of the cases anti-Hbs-Ab were not detectable anymore. Only 1 PLWH had gone through an HBV-infection and cleared, but lost anti-Hbc- and anti Hbs-Ab over time. Chronic hepatitis B infection defined as persistent HBsAg > 6 months could be detected in 7.0% (21/298), all were receiving a TDF- or TAF-containing cART. The vaccination rate in the remaining 207 PLWH was 64.3% (133/207) (Fig. 3). All remaining 74 HBV unvaccinated and infection naïve PLWH received a TDF- or TAF-containing cART.

Vaccination against *Neisseria meningitidis*, which is also part of the EACS vaccination recommendation for PLWH, could be observed in only 9 PLWH(3%). In our evaluation we did not differentiate between the conjugate vaccine which includes protection against A, C W_135_ and Y subtypes and the split vaccine against subtype B which is most commonly used in Germany.

Most likely following infection with *Varizella zoster* during childhood PLWH in our study showed sufficient antibody titers except for 3 cases, who were subsequently vaccinated. All PLWH with positive antibody titers had not undergone vaccination. Their antibodies developed through infection and subsequent immune response.

**Fig. 4:**
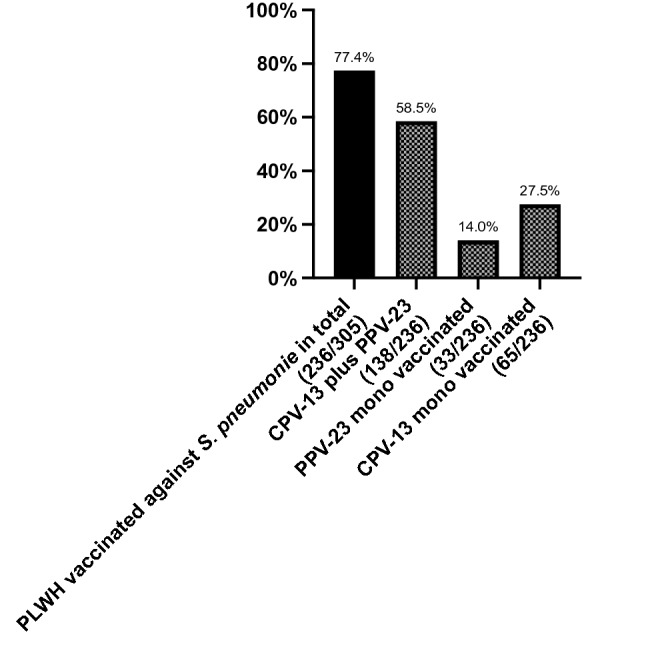
In total, 77.4% (236) of the patients had been vaccinated against Streptococcus pneumoniae. 58.5% (138/236) of them followed the preferred regime with CPV-13 initially and PPV-23 administration at least 6 months after the first dose. 27.5% (65/236) received only one CPV-13 dose without any further vaccination. A general recommendation for a booster dose does not exist to date, though national guidelines are heterogenous

236 PLWH (77.4%) had been vaccinated against Infection with *Streptococcus pneumoniae* with 138 patients (58.5%) receiving the 13-valent vaccine (Prevenar13^®^) and the polysaccharide vaccine (Pneumovax^®^ 23) at some later point, but not before 6 months after the initial dose. Another 33 PLWH (11%) were vaccinated by only using the PPV-23 polysaccharide vaccine (Fig. 4).

## Discussion

Based on our findings implementation of EACS vaccination recommendations among PLWH seems to be partially successful for some of the recommended vaccinations with vaccination rates exceeding the ones in the HIV-negative German population [9–11].

A recent German study published in 2021 analyzed the rates among PLWH older than 50 years for certain vaccinations including Hepatitis A and B, Influenza, *Neisseria meningitidis* and *Streptococcus pneumonia*, as well associated factors for vaccination [12]. The results obtained through this investigation showed similar results for vaccination coverage compared to our study population except for *Neisseria meningitidis* (51.0%), where utilization of vaccination was remarkably higher potentially as a result of a higher average duration of HIV infection and a higher proportion of men who have sex with men in the sample size. Though both studies used questionnaires for evaluation of vaccination rates ours was physician-led and not self-reported as in Drewes et al. which is more prone to biased results. We also consulted patients’ vaccination certificates and medical records. Also our cohort covers a wider age range of PLWH as our participants only had to be older than 18 years and not 50 as in Drewes et al. Taking both main differences together we are confident that our findings provide a more holistic picture of vaccination coverage among PLWH in Germany.

With regard to individual vaccinations we were able to observe a higher vaccination coverage for Hepatitis B immunization compared to the HIV-negative population in Germany, but also compared to other high-risk groups. The vaccination rate in the HIV-negative population is about half the coverage among PLWH in our group with 32.9% [13] vs. 64.3%. Similar results were seen when looking at PLWH living outside of Germany [14]. In 2012 Price et al. screened the UK collaborative HIV cohort (UK CHIC) for Hepatitis B infection and vaccination uptake [15]. The vaccination rate in this survey, which had to be estimated to some degree, reached 58.2%. This is slightly lower compared to our data. Utilization of HBV vaccination in South Brazil, UK or France is similar [14, 15], though in the case of South Brazil the coverage of 57.4% was below the HIV-negative population [16] although the government started to recommend Hepatitis B vaccination for PLWH as well in 2001. In most cases vaccination coverage among PLWH is higher in comparison to the respective HIV-negative population and somewhat lower than rates in seen in our cohort. Yue et al. screened the 2014 and 2015 compiled data from the National Health Interview Surveys (NHIS) for Hepatitis A and Hepatitis B vaccination. Patients with a chronic liver disease which was recorded by a questionnaire reported to have received $\ge$ 1 dose in 35.7% and $\ge$ 2 doses in 29.1% of the cases. The coverage for Hepatitis A vaccine was even a bit lower. 19.4% and 11.5% of the participating patients received $\ge$ 1 and $\ge$ 2 doses of vaccine, respectively [17], which again is below the coverage rate among the PLWH presenting to our HIV clinic. Similar is the higher vaccination rate for Hepatitis B compared to the rates for Hepatitis A.

As far as data are available for vaccination against seasonal influenza in Germany rates were distinctly below the recommended number of 75% in the age group > 60 years [9] and until the season 2017/2018 the already low vaccination rates were even dropping further. With the influenza season 2018/2019, there were first signs of an increase in vaccination rates at levels of 2014/2015 [18]. The subgroup of patients being older than 60 years in our cohort showed a vaccination rate of 83.3% which is compared to some regions in West-Germany more than twice as high. Similar to Bödeker et al. and the *RKI* younger ages are associated with a decrease in vaccination rates [9, 19]. Another cross-sectional survey from Austria investigating coverage of influenza vaccination among PLWH in 2014 found a vaccination rate of 11.9% [20]. In line with findings from other studies, older age was associated with a higher vaccination status. Slightly higher rates for vaccination coverage of pandemic influenza were observed in Greece and France [14, 21] though it seems that vaccination rates across Europe are not achieving recommended goals in general [22]. The coverage of seasonal influenza is higher in our cohort compared to the HIV-negative population as well as former surveys among PLWH, though for the latter adherence to vaccination recommendations seems to be higher. In addition, another reason might be that primary care physicians are more aware of seasonal influenza vaccination than for example vaccination against VZV or *Neisseria meningitidis.* Therefore, the implementation of the EACS guidelines into daily routine might be easier in the case of vaccinations being commonly known among primary care providers (PCP), such as Influenza or HAV and HBV.

The EACS-guidelines recommend vaccination with the conjugated 13-valent vaccine (CPV-13) for all PLWH, independently from a possible vaccination with PPV-23 polysaccharide vaccine at some earlier point in time. A general booster dose is not recommended by EACS, though in some European countries a second dose with PPV-23 is mentioned. This second dose should be given with a least 2 months after the CPV-13 dose. In Germany the application of the second dose should take place between 6 to 12 months after CPV-13 vaccination [23]. Again, with 77.4% we saw a significantly higher rate of vaccination against S. pneumoniae compared to the HIV-negative population, where vaccination is recommend in > 60 years.

In our cohort only 3 patients had been vaccinated against *Varizella zoster (following the assessment)*, as all other participants showed sufficient antibodies after infection, none of them had been vaccinated before.

In Germany HPV vaccination was first introduced in 2007. At that time, it was only recommended for girls, but since 2018 the STIKO also recommends an immunization for males. Nowadays, it is part of the standard protocol for adolescent aged 9 until 14 years. Ideally a vaccination should be done before the age of 18. There are two reasons why none of the PLWH participating in this study were vaccinated against HPV. The vaccines are only licensed for the use in adolescents and subsequently usage of these rather costly vaccines in adults is not reimbursed by German health insurances.

Not only for HPV vaccination, but also for *Neisseria meningitidis* (3.0%) the vaccination rate was too low in our cohort, and the importance of immunization seems to be underestimated. Especially in view of the fact that outbreaks of bacterial meningitis among MSM have recurrently been reported [24–27].

## Conclusions

The implementation of EACS vaccination recommendations among PLWH in daily clinical routine is mostly successful, especially for vaccinations that are widely recommended and administered in the HIV-negative population, such as Influenza, *Streptococcus pneumoniae* or HBV as well. Interestingly, vaccination rates for these three diseases are higher among HIV-infected people than in the HIV-negative German population. On the other hand, there is still room for improvement for some vaccinations, such as VZV, *Neisseria meningitidis* or HPV. Particularly for HPV a clear and comprehensible system of cost coverage for adult patients might be helpful. In addition to that, a nationwide and centralised registration of vaccination rates for example via the electronic health card which is about to be rolled out in Germany might help to recognize gaps in vaccination coverage. This can help to trigger a more coordinated care of PLWH in Germany to achieve higher vaccination rates.
